# Surface N- or O-linked glycans on bovine spermatozoa play minimal role in evading macrophage mediated phagocytosis

**DOI:** 10.3389/fvets.2025.1550100

**Published:** 2025-03-24

**Authors:** Jatinder Singh Chera, Vikrant Gaur, Abhishek Kumar, Fanny Josan, Aditya Patel, Sonam Yadav, Seema Karanwal, Preeti Verma, Vivek Verma, Sushil Kumar, Amit Kumar Bairagi, Sanchi Kamal, Tirtha Kumar Datta, Rakesh Kumar

**Affiliations:** ^1^Animal Genomics Laboratory, Animal Biotechnology Division, ICAR-National Dairy Research Institute, Karnal, India; ^2^Department of Veterinary Gynaecology and Obstetrics, ICAR-National Dairy Research Institute, Karnal, India; ^3^ICAR-Central Institute for Research on Buffaloes, Hisar, India

**Keywords:** phagocytosis, macrophage, spermatozoa, glycocalyx, bovine

## Abstract

Bull spermatozoa possess glycocalyx made of carbohydrate moieties attached to proteins and lipids on their membranes that is involved in fertility associated functions including immune evasion in the female reproductive tract. The current study aimed to establish whether the differences in the glycocalyx of spermatozoa provide selective advantage in evading phagocytosis mediated by female macrophages. Based on removal of either N- or O-linked surface glycans from the spermatozoa, their susceptibility to phagocytosis by macrophages was assessed *in vitro* in bovines (*Bos indicus*) through flow cytometry. We found no significant difference (*p* > 0.05) in the phagocytosis of spermatozoa without N-glycans or O-glycans compared to those with intact glycocalyx. Out of nearly 2,000 events analysed, the mean number of macrophages phagocytosing the spermatozoa were found to be 416, 423 and 345, respectively for spermatozoa with an intact glycocalyx, with N-glycans removed and with O-glycans removed. The difference in the mean values of the individual sample geometric mean fluorescence intensities (*n* = 3) of the phagocytosed spermatozoa among all the treatment groups were also statistically insignificant (*p* > 0.05) indicating that the macrophages are not involved in the selection of spermatozoa based on their surface glycan profiles. Therefore, it is plausible to conclude that macrophages may be exploiting other signature molecules if at all they are involved in the cryptic female choice, or they might be phagocytosing spermatozoa with less stringency that may not be dependent on O- or-N-glycans on sperm surface. However, further studies are required to gain deeper insights into this phenomenon.

## 1 Introduction

The plasma membranes of the mammalian cells possess a polymeric meshwork on their surfaces, known as the glycocalyx which is made of carbohydrates, proteins and lipids (1). Mammalian spermatozoa also possess a thick glycocalyx surrounding their membranes which facilitates interaction with other cells for facilitating fertilization-related processes and survival in the female reproductive tract (FRT). The glycocalyx is formed by several polypeptide chains extending from the sperm membrane onto which carbohydrate moieties are intricately loaded (2, 3). The spermatozoa glycocalyx ranging from 20 to 60 nm in thickness is formed through the synthesis of glycoconjugates in the endoplasmic reticulum and Golgi apparatus during differentiation and maturation (4). The developed spermatozoa thus generated possess a dynamic glycocalyx that enables them to overcome the immunological and physiological barriers of the FRT, undergo capacitation and ultimately fertilize the ovum (5, 6).

The immune response in the FRT is mediated by various immune cells, including phagocytes and lymphocytes (7). Several immune functions such as recognition of pathogen associated molecular patterns (PAMPs) and antibody generation against the pathogens, persistently occur in the FRT (8, 9). However, this immune response can be detrimental to the spermatozoa as they are recognized as ‘non-self’ by the FRT (6). Neutrophils, macrophages and dendritic cells are the phagocytes found in the FRT that can phagocytose spermatozoa (10–12). Successful phagocytosis requires marking of foreign particles for their detection by the professional phagocytes via opsonic, non-opsonic and apoptotic cell receptors (13).

The glycans forming the glycocalyx play essential roles in distinguishing ‘self’ from the ‘non-self’ cells. For example, various glycan binding proteins (GBPs) such as C-type lectin receptors (CLRs), galectins and Siglecs (Sialic acid–binding Ig-like lectins) are present on dendritic cells that function as SAMP (self-associated molecular pattern) receptors by differentiating between self and non-self glycan patterns (14–16). Siglec-1 (Sialoadhesin) on macrophages binds to the sialic acid residues (a SAMP) on cell surfaces, preventing immune response. However, some pathogens possessing sialic acid residues have been reported to undergo phagocytosis by macrophages suggesting the presence of additional, yet unknown, mechanisms for distinguishing ‘self’ from ‘non-self’ (17).

Limited studies have suggested that sialic acid residues protect spermatozoa from macrophage mediated phagocytosis (5, 18). The role of other glycans in shielding the spermatozoa from female macrophages remains an area of investigation. In buffaloes, a lower abundance of O-glycans rendered spermatozoa more susceptible to phagocytosis and NETosis by the PMNs (19). Therefore, the aim of the current study was to decipher whether the macrophages play a similar role in phagocytosing spermatozoa based on their surface glycan profiles in bovines (*Bos indicus*).

## 2 Materials and methods

### 2.1 Selection of animals and blood collection

The Sahiwal cows that had undergone at least one pregnancy throughout their lives were selected for blood collection. The whole blood was collected from the jugular vein into the EDTA vacutainers (BD Biosciences) and brought to the laboratory at room temperature.

### 2.2 Isolation of peripheral blood mononuclear cells (PBMCs)

Prior to PBMC isolation, the following solutions were freshly prepared; 54% Iodixanol made by mixing 5.4 volumes of OptiPrep (Serumwerk) and 0.6 volumes of 8.5% NaCl (Bio Basic), density barrier of 1.076 gm/mL by mixing 1 volume of OptiPrep and 3 volumes of complete RPMI medium containing RPMI1640 (61870036, Gibco) and 10% FBS (16140071, Gibco). In a tube, 5 mL blood was mixed with 1 mL of 54% Iodixanol. Above the mixture, 6 mL of 1.076 gm/mL density barrier was layered gently and another layer of 500 μL complete RPMI was also gently added on top of the density barrier. The tube was centrifuged in a refrigerated centrifuge (Hermle Z 326 K) at 700*g* for 30 min at 4°C without brakes. The top layer was taken and mixed with 2 volumes of RPMI and again centrifuged at 1,000*g* at 4°C for 7 min. The pellet was resuspended in RPMI and the cells were counted before proceeding for cell culture. The cells were counted through flow cytometry (BD Accuri C6 Plus) by running limited volume (10 μL) with necessary gates applied (Supplementary Figure S1). The detailed flow cytometry protocol is described later in this section.

### 2.3 *In vitro* generation of monocyte derived macrophages (MDMs)

Approximately, 0.2 × 10^6^ monocytes were seeded in each well of 12-well cell culture plate (Nunc) in RPMI+10% FBS + 1X Antibiotic-antimycotic (15240062, Gibco). The cells were incubated for 24 h in humidified CO_2_ incubator (NU-8600E, Nuaire) with 5% CO_2_. The media was then discarded and washed with 1X PBS before replacing with RPMI+10% FBS + 1X Antibiotic-antimycotic and 100 ng/mL M-CSF (RP1614H, Kingfisher Biotech). The cells were cultured for ~5–7 days with replacement of media with fresh RPMI+M-CSF after every 2 days till the macrophages were observed under the microscope (Supplementary Figure S2).

### 2.4 Processing of frozen bovine semen straws

Semen straws of healthy Sahiwal bulls were procured from Artificial Breeding Research Centre, National Dairy Research Institute, Karnal. For one experiment, 3 straws were thawed for 1 min in water maintained oreat 37°C and the semen was washed in non-capacitating media (NCM) prepared by adding 155 μL of 60% sodium lactate (L4263, Sigma) and 5.5 mg of sodium pyruvate (P5280, Sigma) in 1X PBS upto a final volume of 50 mL. Approximately, 4 volumes of the NCM were added and centrifuged thrice using a fixed angle rotor at 300*g* for 5 min at 37°C and the pellet was resuspended in 200 μL NCM. A drop of semen was mounted on a glass slide to visually assess the motility. A dilution of 1:100 was also prepared to count the number of spermatozoa using flow cytometry.

### 2.5 Enzyme based removal of N-linked and O-linked glycans from spermatozoa

Approximately, 2 × 10^6^ spermatozoa were taken in 19 μL of NCM for glycan removal. For N-linked glycan removal, 500 units of PNGase F (P0704, New England Biolabs) was added and incubated for different time points (1, 2 and 4 h) at 37°C in a humidified incubator (NU-8600E, Nuaire). For removal of core 1 and core 3 O-linked glycans, the spermatozoa were treated with 50 units of α2-3,6,8,9 Neuraminidase A (P0722, New England Biolabs) and 60,000 units of O-glycosidase (P0733, New England Biolabs) for different time points (1, 2 and 4 h) at 37°C in a humidified incubator (NU-8600E, Nuaire). The level of deglycosylation was confirmed by FITC labelled ABL (*Agaricus bisporus* lectin) and FITC labelled Jacalin in case of O-linked glycans while FITC labelled LEL (*Lycopersicon esculentum* lectin) was used for the N-linked glycans (19). These lectins were procured from Vector Laboratories, United States. The optimal concentration was determined in spermatozoa for clearly separating the signal from noise (Supplementary Figure S3). For lectin binding assays, the concentrations of 40, 30, and 50 μg/mL were selected for Jacalin-FITC, ABL-FITC, and LEL-FITC, respectively.

### 2.6 Flow cytometry for cell counting, data acquisition and visualization

The BD Accuri C6 plus flow cytometer was calibrated daily with 8 and 6 peak calibration beads while the QC was performed every second day using BD CS&T RUO beads. A threshold of 80,000 events in the forward scatter (FSC) was used to remove very small events during the data acquisition. The flow rate was set to low (14 μL/min) for sample analysis while for cell counting, the flow rate was set to fast (66 μL/min). For all the flow cytometer analyses, the events were first plotted on FSC vs. SSC (side scatter) to gate out the debris and specifically for sample analysis using fluorochromes, FSC-A (area) vs. FSC-H (height) plot was used on the events that were obtained in FSC vs. SSC plot to gate out the doublets (Supplementary Figure S4). For FITC dye, FL1 bandpass filter (533/30 nm) and for eFluor670, FL4 bandpass filter (675/25 nm) were used to acquire the data. The data files obtained were analyzed using FlowJo v10.8.1. The data plots were also generated through FlowJo.

### 2.7 Assessment of phagocytosis of spermatozoa by monocyte derived macrophages

For phagocytosis assay, 1.5 × 10^6^ spermatozoa were initially labelled with 2 μM eFluor 670 dye (65-0840-90, Invitrogen) in 1X PBS (20). The spermatozoa were incubated in the eFluor 670 solution for 10 min in the dark at room temperature. The spermatozoa were washed 3 times with 1X PBS for 5 min each at 300*g* at room temperature. The labelled spermatozoa were again counted and approximately 0.4 × 10^6^ spermatozoa were co-incubated with monocyte derived macrophages in the culture plate containing RPMI media for 1 h at 37°C in humidified CO_2_ incubator (maintaining a ratio of 1:2 between the cells and spermatozoa). The cells were then washed 3 times with 1X PBS and trypsinized. Trypsin was inactivated with complete RPMI media followed by washing with 1X PBS. The cell pellet was then resuspended in ice cold 1% BSA (prepared in 1X PBS) to a concentration of 10^7^ cells per mL. The cells were then labelled with FITC-anti CD14 antibody (MA1-82074, Invitrogen) in a dilution of 1:10 and incubated in dark at 4°C for 30 min. The cells were then washed 3 times with 2 mL ice cold 1% BSA and then resuspended in ice cold 1X PBS. Phagocytosis was then assessed through flow cytometry. The phagocytosis of spermatozoa was also visualized under a bright field microscope (Supplementary Videos 1–3).

### 2.8 Statistical analysis

The flow cytometry data for individual experiments (*n* = 3) were statistically analyzed through GraphPad Prism v8.0 using one way ANOVA (Analysis of Variance) along with Tukey’s *post hoc* multiple comparison tests. The analyzed data were plotted as histograms using GraphPad Prism.

## 3 Results

### 3.1 O-linked glycans are not removed from the surface of spermatozoa when treated with O-glycosidase alone

**Figure 1:**
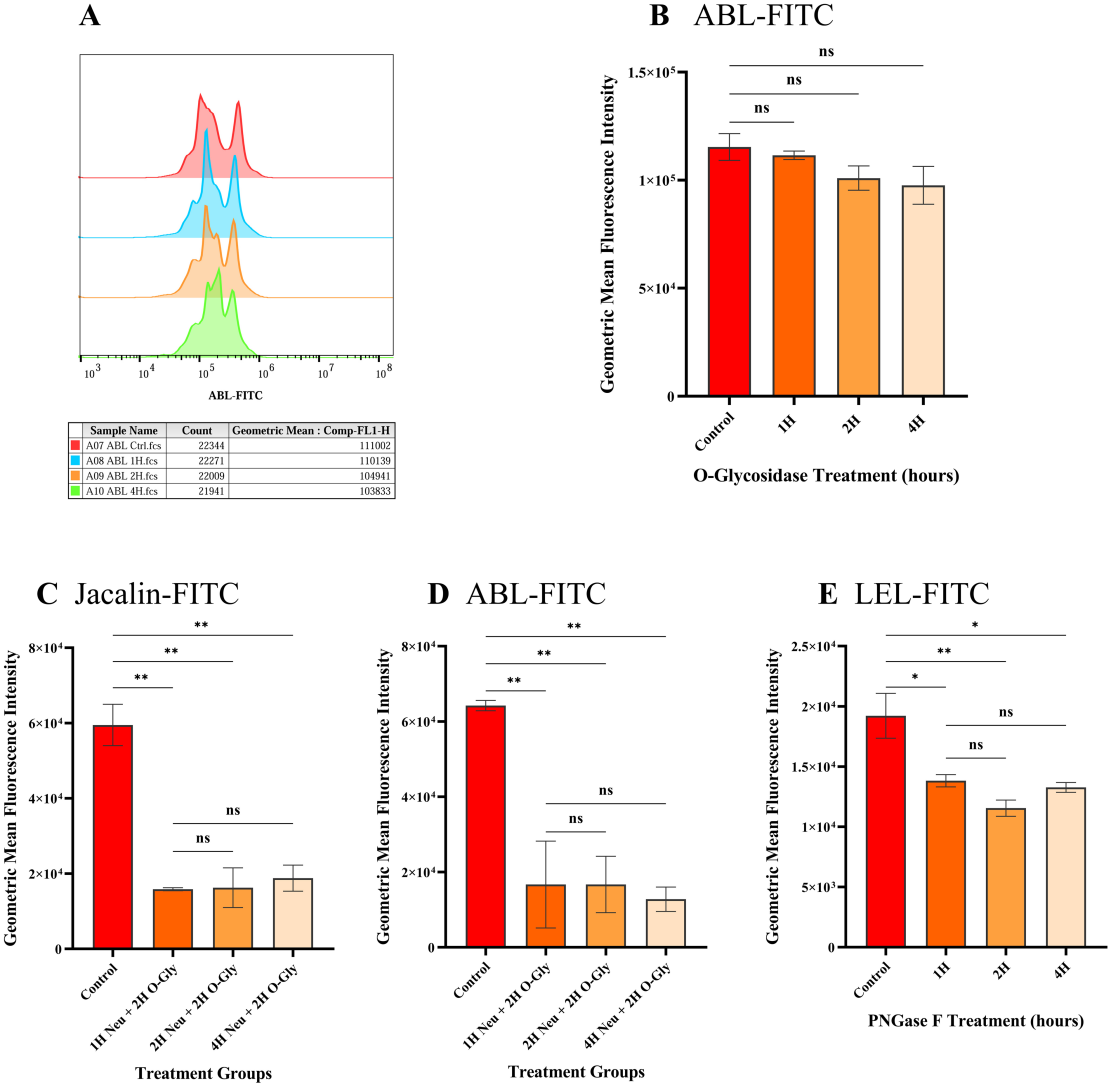
Geometric mean fluorescent intensities (GMFIs) of FITC labelled lectins bound to spermatozoa after treatment with different glycanases to remove either N- or O-linked glycans. **(A)** Histogram representing the cellular frequency distribution as peaks with GMFIs of ABL-FITC on the x-axis. The table below the histogram indicates the sample type and GMFIs at different time points where ‘H’ after the numeric value denotes time in hours. **(B-E)** Comparison of GMFIs of spermatozoa treated with different glycan removing enzymes at 1, 2 and 4 h. Data are presented as the means of individual GMFIs recorded from biological replicates (*n* = 3) with standard deviation shown as error bars. **(B)** GMFIs of ABL-FITC bound to spermatozoa treated with O-glycosidase alone. **(C)** GMFIs of Jacalin-FITC bound to spermatozoa treated with α2-3,6,8,9 Neuraminidase A (Neu) and O-glycosidase (O-Gly) to remove O-glycans. **(D)** GMFIs of ABL-FITC bound to spermatozoa treated with α2-3,6,8,9 Neuraminidase A (Neu) and O-glycosidase (O-Gly). **(E)** GMFIs of LEL-FITC bound to spermatozoa treated with PNGase F to remove N-glycans. Asterisks over the bars represent the level of significance at 95% CI between control and treated groups while ‘ns’ denotes non-significant as determined by one-way ANOVA along with Tukey’s *post hoc* multiple comparison test (*n* = 3). The values of significance are: 0.1234 (ns), 0.0332 (*), 0.0021 (**), 0.0002 (***) and < 0.0001 (****).

To assess the removal of O-linked glycans, spermatozoa were treated with O-glycosidase and incubated with ABL-FITC at different time points (1, 2, and 4 h). Time points beyond 4 h were not considered to avoid capacitation that could potentially lead to modification of surface glycans. There was no significant difference in the geometric mean fluorescence intensities (GMFI) of O-glycosidase-treated spermatozoa compared to the untreated spermatozoa at any time point (Figures 1A,B). This suggests that O-glycosidase alone is insufficient for efficient removal of O-glycans from spermatozoa.

### 3.2 Treatment with α2-3,6,8,9 neuraminidase a prior to O-glycosidase is crucial for removal of O-glycans from sperm surface while N-linked glycans were removed by PNGase F

O-linked glycans were effectively removed by pre-treating spermatozoa with α2-3,6,8,9 Neuraminidase A for 1 h, followed by O-glycosidase for 2 h. This treatment resulted in a significant reduction (*p* < 0.05) in the geometric mean fluorescence intensities (GMFIs) of ABL-FITC and Jacalin-FITC. The average GMFI value (*n* = 3) of Jacalin-FITC and ABL-FITC in untreated spermatozoa were 5.95 × 10^4^ and 6.42 × 10^4^, respectively, while in the spermatozoa treated with Neuraminidase A for 1 h and O-glycosidase for 2 h, it was 1.58 × 10^4^ and 1.67 × 10^4^ (Figures 1C,D and Supplementary Figures 5A,B). Increasing the incubation time of Neuraminidase A treatment to either 2 h or 4 h did not have any further significant effect on the GMFI values (*p* > 0.05). This indicates that the terminal sialic acids likely prevent the cleavage of core O-glycans from O-glycosidases. Hence, the treatment with Neuraminidase A for 1 h followed by O-glycosidase for 2 h was selected for removal of O-glycans prior to phagocytosis assay.

Spermatozoa were also treated with PNGase F for 1, 2 and 4 h to remove surface N-linked glycans. The removal of the N-glycans was assessed through the binding of FITC labelled LEL lectin. It was observed that the GMFI of LEL-FITC was significantly reduced as early as 1 h post treatment with PNGase F and a maximum significant reduction was observed at 2 h post treatment (Figure 1E and Supplementary Figure 5C). The average GMFI value of untreated spermatozoa was 1.92 × 10^4^ while it was reduced to 1.38 × 10^4^ (*p* = 0.0224) and 1.15 × 10^4^ (*p* = 0.0063) after 1 and 2 h of PNGase F treatment, respectively and no significant difference was observed between the GMFIs after 2 and 4 h (*p* > 0.05). Therefore, 2 h of PNGase F treatment was selected for phagocytosis assay.

### 3.3 Macrophages do not exhibit bias in phagocytosing spermatozoa with intact or altered surface glycans

**Figure 2:**
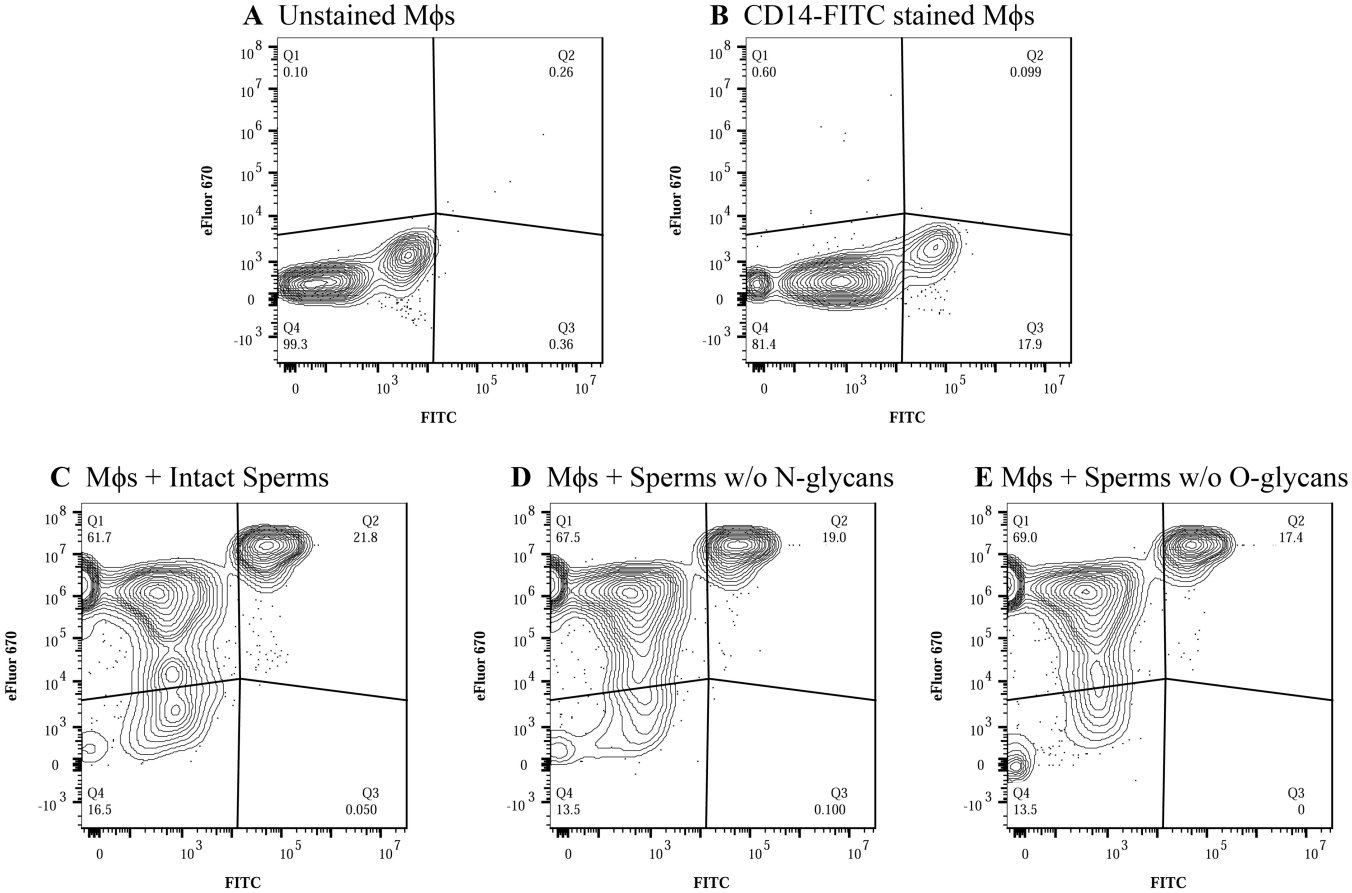
Flow cytometric analysis of macrophage mediated phagocytosis of spermatozoa. Contour plots were generated in FlowJo at a probability of 5%, including outliers. The quadrants denote the following: Q1-Stained spermatozoa, Q2-CD14+ macrophages phagocytosing spermatozoa, Q3-CD14+ macrophages and Q4-Unstained cells. The numerical values below each quadrant label indicate the percentage of events within that quadrant. Approximately 2,000 events were analyzed per sample. The x- and y-axes represent the logarithmic fluorescence intensities of CD14-FITC and eFluor 670, respectively. **(A)** Unstained macrophages, **(B)** Macrophages stained with CD14-FITC, **(C)** Stained macrophages incubated with spermatozoa with intact glycocalyx, **(D)** Stained macrophages incubated with spermatozoa lacking surface N-glycans. **(E)** Stained macrophages incubated with spermatozoa lacking surface O-glycans. Contour plots for the stained and unstained spermatozoa are provided in the Supplementary Figure S6.

**Figure 3:**
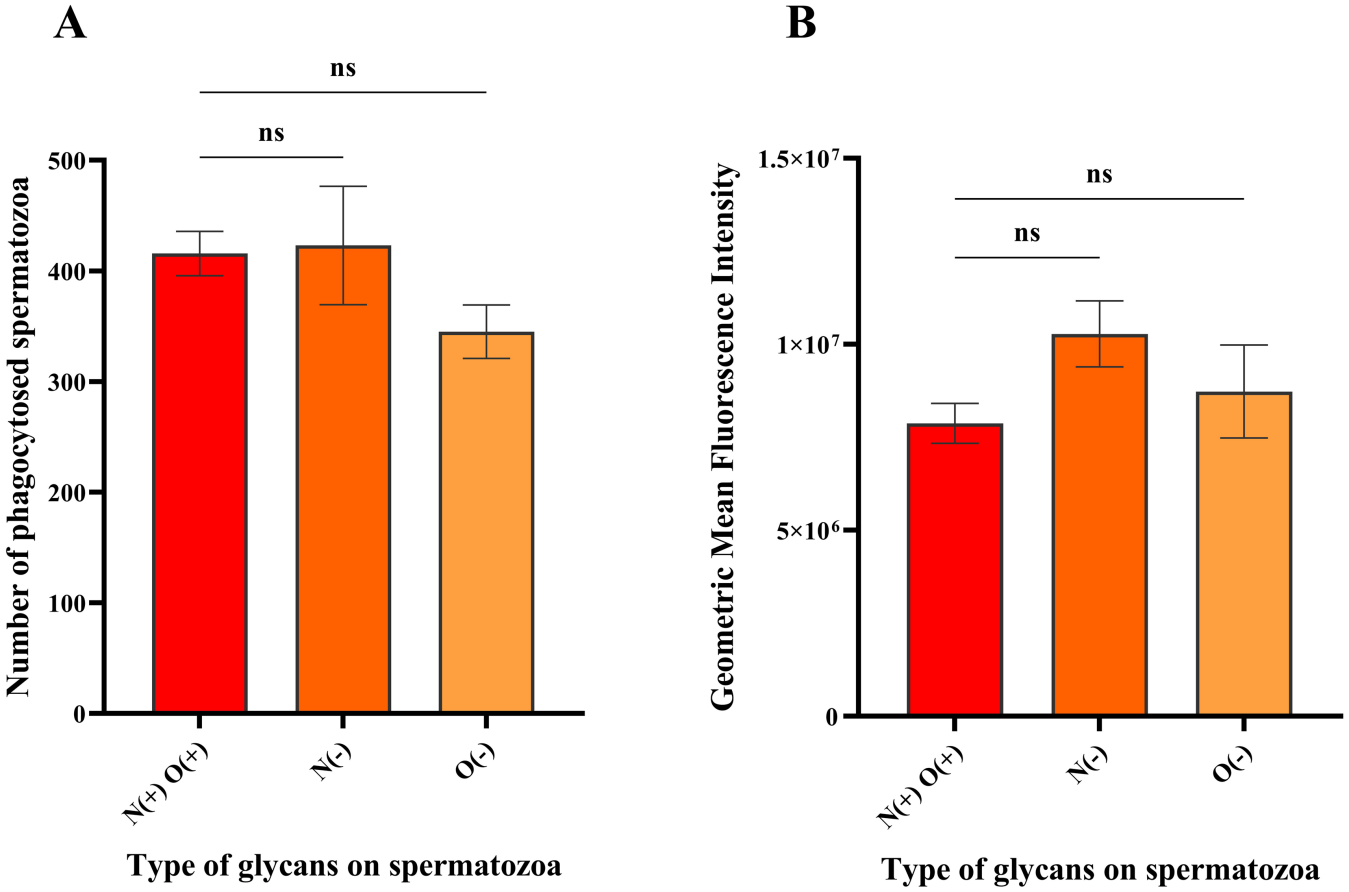
Histograms depicting the mean population and geometric mean fluorescence intensities (GMFIs) of the macrophages phagocytosing spermatozoa with different glycan profiles. **(A)** Mean of the events from the Q2 of the contour plots in Figures 2C–E (*n* = 3) representing the macrophages interacting with spermatozoa having intact glycocalyx, without N-glycans and without O-glycans, respectively. **(B)** Mean GMFIs of the replicates for each sample type (*n* = 3) in the quadrant Q2 of Figures 2C–E wherein, N(+)O(+) denote intact glycocalyx, N(−) denotes without N-glycans and O(−) denotes without O-glycans. The ‘ns’ denotes non-significant as determined by one-way ANOVA along with Tukey’s post hoc multiple comparison test (*p* > 0.05).

Phagocytosis of the spermatozoa was assessed using quadrant analysis of contour plots generated in FlowJo using the logarithmic fluorescent intensities of fluorochromes eFluor 670 and FITC (CD14). Unstained and CD14-FITC stained macrophages served as controls (Figures 2A,B) along with spermatozoa controls that were unstained or labelled with eFluor 670 (Supplementary Figure S6). In CD14 labelled samples, around 17.9% of events fell in quadrant Q3 (Figure 2B) indicating the CD14+ macrophage population. The mean number of events in quadrant Q2 (macrophages phagocytosing spermatozoa) were 416, 423 and 345 for spermatozoa with intact glycocalyx, without N-glycans and without O-glycans, respectively (Figures 2C–E, 3A, *n* = 3). While phagocytosis was lowest for spermatozoa lacking O-glycans, the differences among all groups were not statistically significant (*p* > 0.05).

Additionally, the geometric mean fluorescence intensities of the events recorded in Q2 were compared, with average values of 7.88 × 10^6^, 1.03 × 10^7^ and 8.73 × 10^6^ for spermatozoa with intact glycocalyx, without N-glycans and without O-glycans, respectively (Figure 3B, *n* = 3). The GMFIs among all the samples were also found to be insignificant (*p* > 0.05) indicating that there was no difference in the level of macrophage mediated phagocytosis against spermatozoa with either intact surface glycocalyx or with N-glycans and O-glycans removed.

## 4 Discussion

The current study aimed to elucidate the role of core N- and O-linked glycans on the surface of spermatozoa in modulating their susceptibility to phagocytosis by macrophages. This was achieved by deglycosylating the spermatozoa using specific enzymes. The efficient removal of O-glycans from spermatozoa requires treatment with both, Neuraminidase (to remove terminal sialic acids) and O-glycosidase indicating that the terminal sialic acid residues protect the core O-glycans from O-glycosidases. PNGase F efficiently removes N-linked glycans efficiently from the surface of spermatozoa. The interaction between the spermatozoa and immune cells, particularly phagocytes, plays a crucial role in reproductive biology and fertility (21).

While previous research has highlighted the significance of glycosylation patterns on spermatozoa in evading female immune responses including phagocytosis, our findings suggest that N-linked and O-linked glycans on the sperm surface (thawed from frozen straws) may not significantly affect their vulnerability to macrophage-mediated phagocytosis. Among phagocytes, the role of neutrophils in the female reproductive tract (FRT) has been extensively studied in context to cryptic female choice. Neutrophils are the pre-dominant phagocytes populating the FRT during the oestrous phase, i.e., the stage of estrus cycle when semen is deposited in the vagina or uterus either through mating or artificial insemination (22–25). Although neutrophils do not exhibit any change in their interactions with spermatozoa upon modification of simple sugars, they significantly increase the phagocytosis of spermatozoa lacking membrane associated O-linked glycans (19, 26). Sialic acids are known to protect mammalian cells by shielding signature motifs from opsonins thereby, preventing phagocytosis (18, 27). During enzymatic removal of core O-linked glycans, terminal sialic acid residues are also cleaved off particularly by neuraminidase, along with other sugars. Under these conditions, it was expected that macrophage mediated phagocytosis of frozen–thawed spermatozoa would increase significantly. However, no difference was observed in the phagocytosis rate of spermatozoa, regardless of whether they possess intact glycocalyx, lacking N-glycans or O-glycans. This observation contrasts with a previous report showing that sialic acids on spermatozoa provide protection against macrophage mediated phagocytosis (5). However, the sperms used in this study were epididymal in nature which are immature compared to fully developed spermatozoa. Additionally, phagocytosis of epididymal spermatozoa increased only when they were supplemented with seminal vesicle fluid and sialidase enzyme. There was no significant increase in phagocytosis when only spermatozoa were treated with sialidase (without seminal vesicle fluid). Our findings clearly highlight that the macrophages are not specialized to selectively phagocytose defective spermatozoa, as seen in the case of PMNs like neutrophils. This may be because, the neutrophils are the most active phagocytes that populate the female reproductive tract (77%–86%) while macrophages and T-lymphocytes are much fewer in number (2%–10.6%) after sperm entry (28) and partly, due to the prolonged duration for differentiation of monocytes into macrophages.

A previous report in boars revealed that after oestrous detection followed by artificial insemination triggered a massive influx of PMNs into the FRT (92%–99%). Similarly, in sows, neutrophils were predominant leukocytes populating the endometrium during the early stages of oestrus and pro-estrus (29, 30). Macrophages, on the other hand, appear in early dioestrus likely to phagocytose and remove dead cells (29). In humans, macrophages in the FRT predominantly phagocytose post-capacitated spermatozoa several hours after coitus (12). In ewes, neutrophils peak at 6 h post mating while macrophages peak at 18–48 h post mating particularly, in the uterus and mid-uterine horns where spermatozoa undergo capacitation (31). Another crucial function of macrophages in the uterus is to facilitate implantation, decidual formation and placenta development that occur at much later stages following successful fertilization (32–34). Therefore, while neutrophils play a critical role in selecting the best spermatozoa immediately after mating, macrophages seem to function at a later stage. Although, specific glycans help spermatozoa evade phagocytosis, this is not their sole function. Various selection strategies apart from immune attack by the female cells, such as cervical mucus penetration, sperm oviduct binding, etc. are also crucial. For example, beta-defensin 126 on the sperm surface is highly O-glycosylated with sialic acids, which is essential for penetrating thick cervical mucus (35). Additionally, beta defensin 126 helps shield sperm surface antigens from the female immune system (36). Removal of sialic acids from beta-defensin 126 has also been shown to impair sperm-oviduct epithelium binding, a process necessary for synchronous capacitation and successful fertilization (37, 38).

Taken together, it can be concluded that macrophages do not exhibit a preference for phagocytosing frozen–thawed spermatozoa based on their surface glycan profiles. However, there are certain limitations in the study. We primarily focused on perturbations of N-linked and O-linked glycans based on previous report, but other glycans may also play a role in macrophage mediated phagocytosis. Although, frozen thawed semen was used in the study which is the prime requirement of artificial insemination, the use of spermatozoa from fresh semen may better mimic the *in vivo* biological conditions. Additionally, specific blocking of glycans on the sperm surface could provide insight into their interactions with macrophage glycan binding proteins. In this study, we employed an isolated *in vitro* culture model to assess macrophage-sperm interactions whereas, development of *in vivo* study or multi-cell model would offer a comprehensive representation of the overall immune responses generated by macrophages in the FRT. Future studies are warranted to address these limitations and provide a better understanding of macrophage mediated phagocytosis against spermatozoa with modified glycocalyx.

## Data Availability

The original contributions presented in the study are included in the article/Supplementary material, further inquiries can be directed to the corresponding author.

## Supplementary material

SUPPLEMENTARY VIDEO 1 Live macrophage mediated phagocytosis of spermatozoa with intact surface glycocalyx.

SUPPLEMENTARY VIDEO 2 Live macrophage mediated phagocytosis of spermatozoa without surface N-glycans.

SUPPLEMENTARY VIDEO 3 Live macrophage mediated phagocytosis of spermatozoa without surface O-glycans.
