# Supplementary material for: Surface N- or O-linked glycans on bovine spermatozoa play minimal role in evading macrophage mediated phagocytosis
Source: Front Vet Sci. 2025 Mar 24;12:1550100. doi: 10.3389/fvets.2025.1550100 (PMC11973392; doi:10.3389/fvets.2025.1550100)
Supplement: Supplementary file 4 [file Data_Sheet_1.docx]

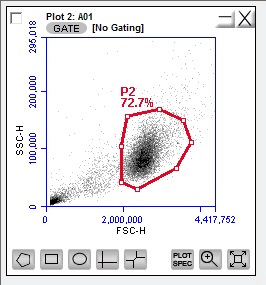


***Figure S1. Cell counting through flow cytometry after monocyte isolation.*** The method employed for monocyte isolation yielded above 70% monocyte population with little lymphocyte contamination.


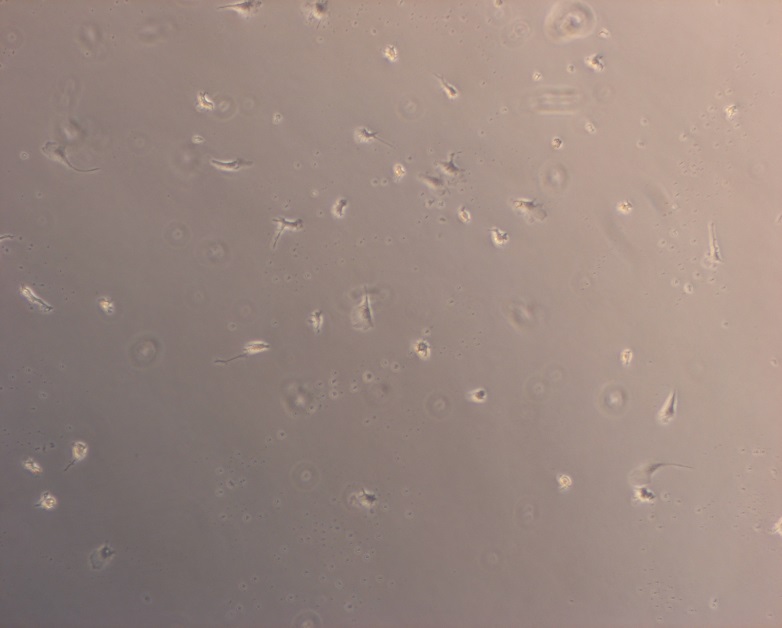


***Figure S2. Monocyte culture and differentiation into macrophages.*** The monocytes were seeded on the 12 well culture plate and allowed to differentiate into macrophages. The image was captured on day 6 since monocyte seeding in the plate and replacement with fresh complete RPMI media and M-CSF after every 2 days was conducted. The round monocytes started exhibiting extended cytoplasm that is a characteristic of macrophages.


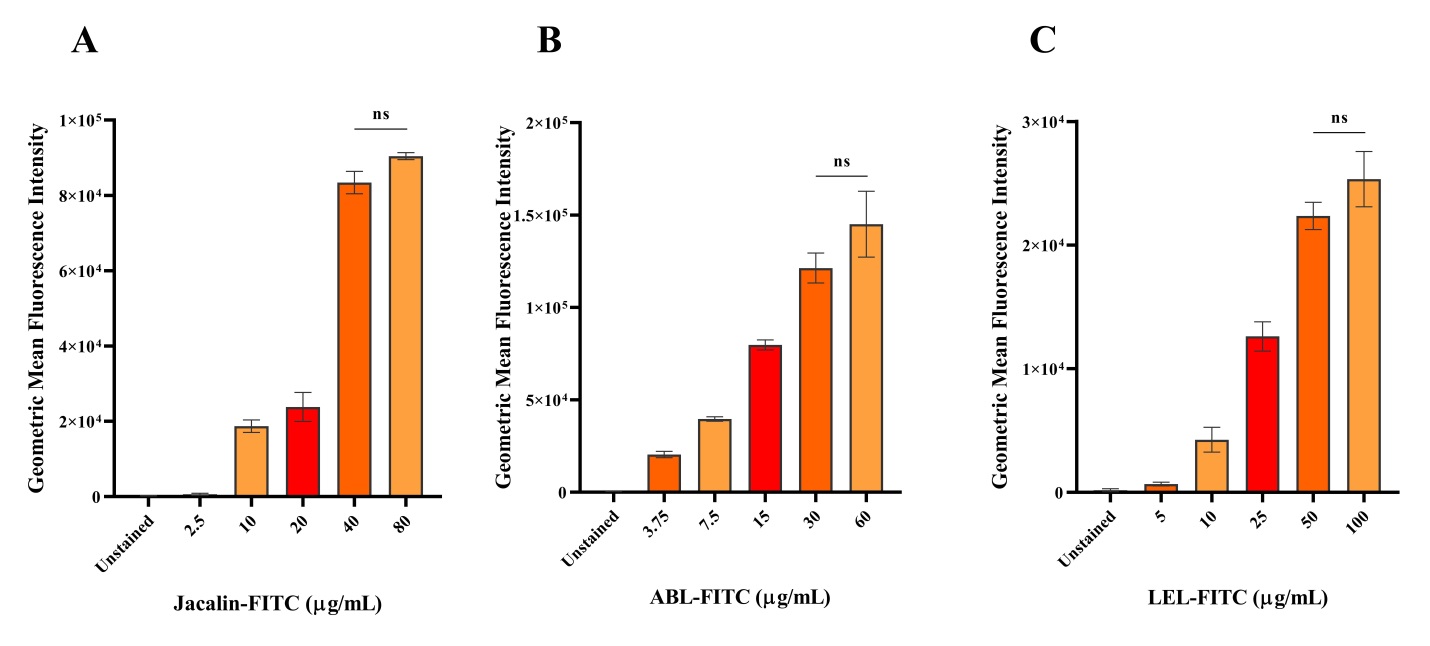
***Figure S3. Determination of optimal concentration of FITC labelled lectins for spermatozoa surface glycan detection.*** The figures depict the concentration ranges (x-axes) and the GMFI values (y-axes) for **A.** Jacalin-FITC, **B.** ABL-FITC and **C.** LEL-FITC bound to spermatozoa of Sahiwal bulls. The ‘ns’ denotes non-significant difference between the treatment groups at 95% C.I as per the one-way ANOVA along with Tukey’s post hoc multiple comparison test (p>0.05). The concentration at which the difference between the mean GMFIs became non-significant compared to the next higher concentration was selected for the lectin binding assays (saturation point).


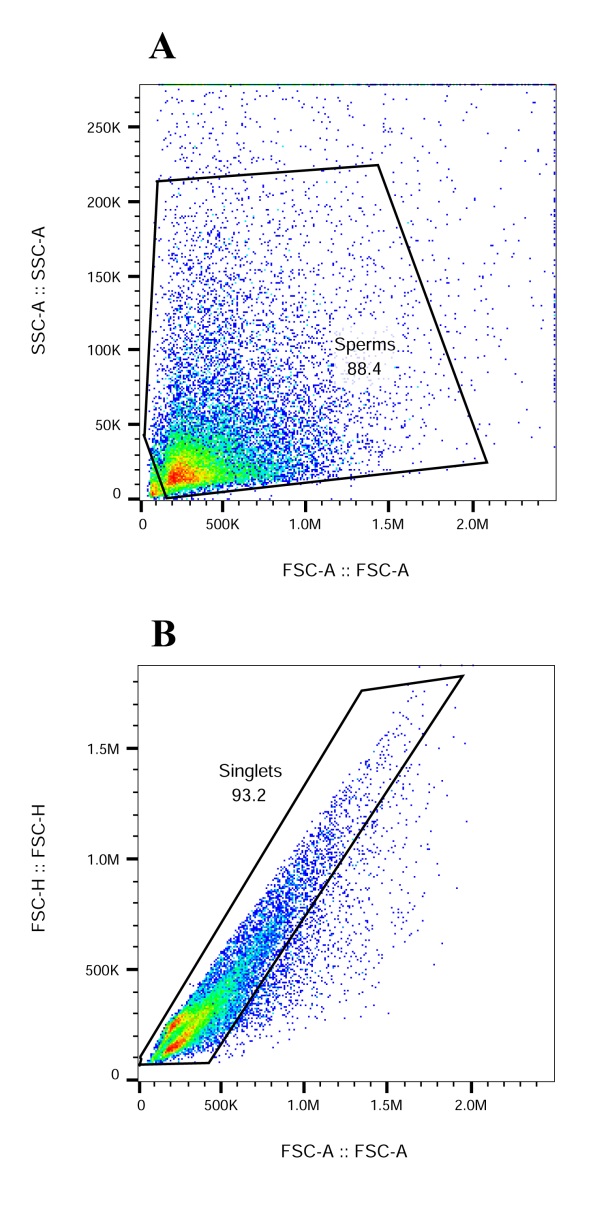


***Figure S4. Gating strategies for flow cytometry to remove debris and doublets.* A.** The density plot between FSC-A and SSC-A allowed to gate out debris from the analysis. These gated populations were subsequently used for another gating strategy to select on singlets (shown in the next figure) **B.** The density plot between FSC-H and FSC-A made it possible to select only the single cells from the population by gating out the doublets. The density plots shown in the figure were generated for sperm samples and similar gating strategies were also employed for monocyte derived macrophages as well. The data visualization was carried out using FlowJo (BD Biosciences).


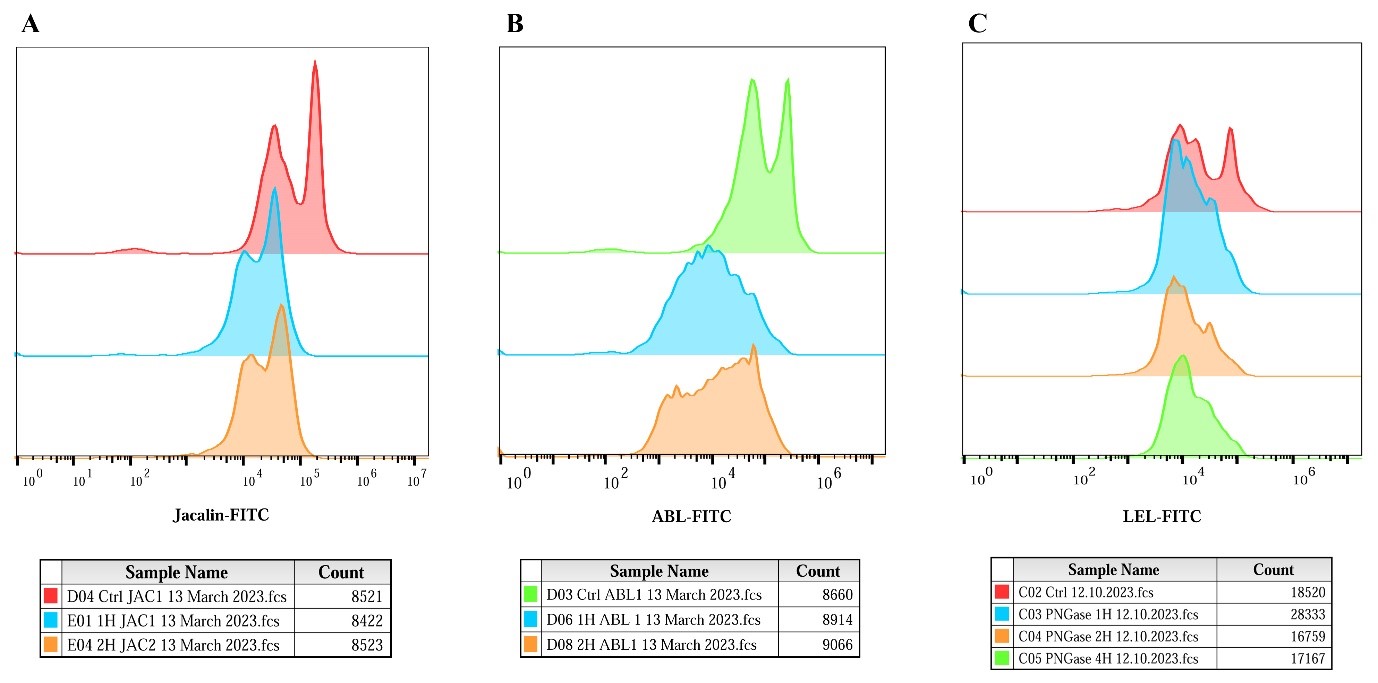


***Figure S5.*** ***The histograms representing the cellular frequency distribution in the form of peaks with respect to geometric mean fluorescent intensities (GMFIs) of FITC labelled lectins.*** The spermatozoa were treated with α2-3,6,8,9 Neuraminidase A and O-glycosidase to remove O-linked glycans followed by labelling with **A.** Jacalin-FITC and **B.** ABL-FITC. **C.** represents the spermatozoa treated with PNGase F for 1 hour, 2 hours and 4 hours to remove N-linked glycans followed by labelling with LEL-FITC (the table below each histogram mentions the type of sample at different time points where ‘H‘ after the numeric value represents time in hours).


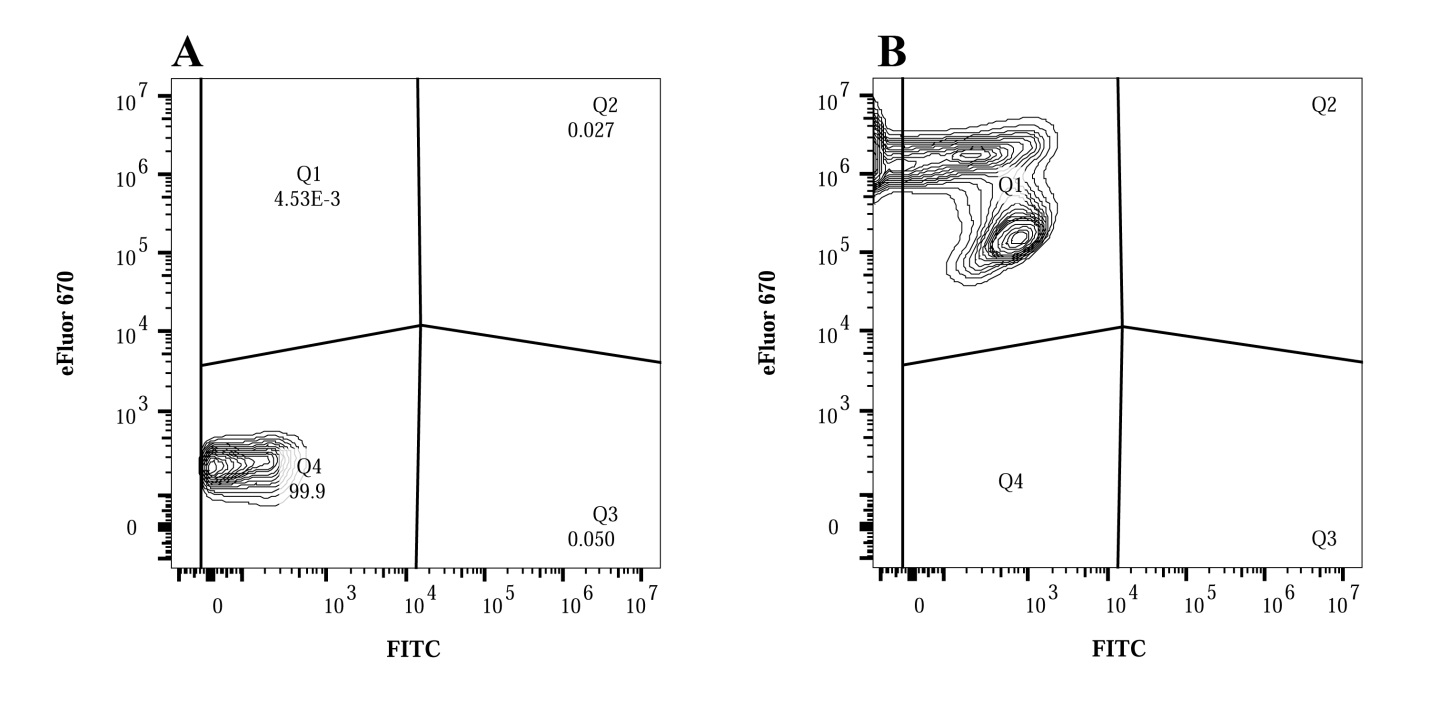
***Figure S6.*** ***Spermatozoa controls for flow cytometry-based phagocytosis detection.*** The contour plots were generated in Flowjo at a probability of 5% along with outliers. The quadrants denote the following: Q1- Stained spermatozoa, Q2- CD14+ macrophages interacting/phagocytosing spermatozoa, Q3- CD14+ macrophages and Q4- Unstained cells. The x-axes and the y-axes represent the logarithmic fluorescence intensities of CD14-FITC and eFluor 670 respectively. **A.** Sample containing unstained spermatozoa and **B.** Sample containing spermatozoa stained with eFluor 670.


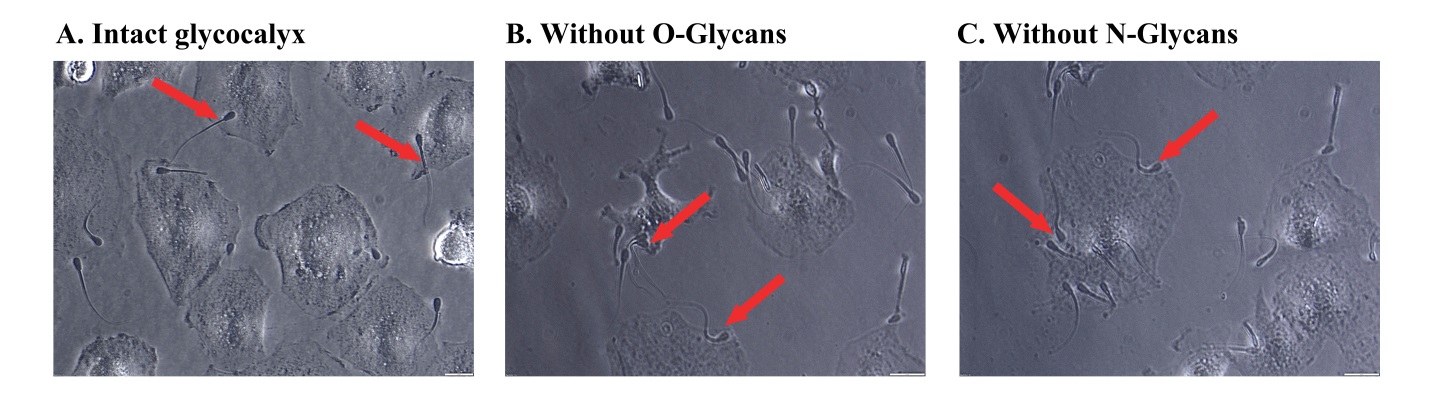
***Figure S7.*** ***Macrophage mediated phagocytosis of spermatozoa.*** The images directly captured from the culture plate show live macrophage mediated phagocytosis of **A.** spermatozoa with intact glycocalyx, **B.** spermatozoa without O-glycans and **C.** spermatozoa without N-glycans as observed through bright field microscope at 60x magnification (additional 10x magnification from eye-piece). The red arrows indicate spermatozoa captured by the macrophages.


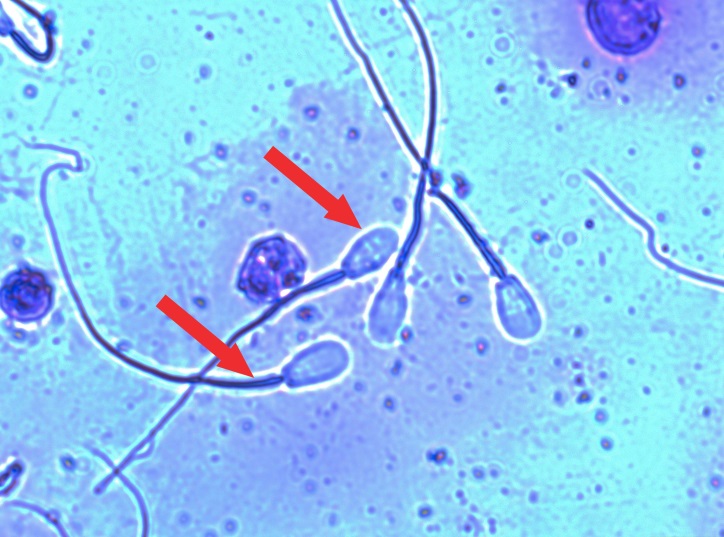


***Figure S8.*** ***Giemsa-stained images of macrophage phagocytosing spermatozoa.***  Image of macrophage-spermatozoa interaction captured after Giemsa staining on a slide and observed at 100X magnification (additional 10X for eye-piece). The red arrows indicate spermatozoa captured by the macrophages.
